# Enhancing Metabolomic Coverage in Positive Ionization Mode Using Dicationic Reagents by Infrared Matrix-Assisted Laser Desorption Electrospray Ionization

**DOI:** 10.3390/metabo11120810

**Published:** 2021-11-29

**Authors:** Ying Xi, David C. Muddiman

**Affiliations:** 1 FTMS Laboratory for Human Health Research, Department of Chemistry, North Carolina State University, Raleigh, NC 27695, USA; yxi3@ncsu.edu; 2 Molecular Education, Technology and Research Innovation Center (METRIC), North Carolina State University, Raleigh, NC 27695, USA

**Keywords:** metabolites, dicationic reagent, ambient ionization, IR-MALDESI, mass spectrometry imaging

## Abstract

Mass spectrometry imaging is a powerful tool to analyze a large number of metabolites with their spatial coordinates collected throughout the sample. However, the significant differences in ionization efficiency pose a big challenge to metabolomic mass spectrometry imaging. To solve the challenge and obtain a complete data profile, researchers typically perform experiments in both positive and negative ionization modes, which is time-consuming. In this work, we evaluated the use of the dicationic reagent, 1,5-pentanediyl-bis(1-butylpyrrolidinium) difluoride (abbreviated to [C_5_(bpyr)_2_]F_2_) to detect a broad range of metabolites in the positive ionization mode by infrared matrix-assisted laser desorption electrospray ionization mass spectrometry imaging (IR-MALDESI MSI). [C_5_(bpyr)_2_]F_2_ at 10 µM was doped in 50% MeOH/H_2_O (*v*/*v*) electrospray solvent to form +1 charged adducted ions with anionic species (−1 charged) through post-electrospray ionization. This method was demonstrated with sectioned rat liver and hen ovary. A total of 73 deprotonated metabolites from rat liver tissue sections were successfully adducted with [C_5_(bpyr)_2_]^2+^ and putatively identified in the adducted positive ionization polarity, along with 164 positively charged metabolite ions commonly seen in positive ionization mode, which resulted in 44% increased molecular coverage. In addition, we were able to generate images of hen ovary sections showing their morphological features. Following-up tandem mass spectrometry (MS/MS) indicated that this dicationic reagent [C_5_(bpyr)_2_]^2+^ could form ionic bonds with the headgroup of glycerophospholipid ions. The addition of the dicationic reagent [C_5_(bpyr)_2_]^2+^ in the electrospray solvent provides a rapid and effective way to enhance the detection of metabolites in positive ionization mode.

## 1. Introduction

Metabolomics plays a critical role in understanding human biology regarding molecular functions and cellular activities [1]. Comparing metabolic differences between normal and abnormal pathways could contribute to our knowledge about metabolites’ properties and their involvement in different stages of disease processes [2,3]. Additionally, the abundances of metabolites, as well as their spatial distributions across biological systems, provide insights into novel biomarkers useful for disease prognosis and diagnosis [3,4,5], which may promote personalized cancer treatment [1,3,6]. Therefore, it is important and necessary to achieve a comprehensive metabolite profile in a high-accuracy and high-throughput manner.

Mass spectrometry imaging (MSI) is one of the most effective analytical approaches to measuring metabolites qualitatively and quantitatively due to its ability to simultaneously detect hundreds to thousands of analytes based on their respective mass-to-charge ratio (*m*/*z*) and abundance. In the meantime, the exact coordinates where these metabolites are within the sample can be recorded in MSI datasets. The abundances of analyte(s) combined with their corresponding locations can be used to generate ion heat maps, which are especially helpful in discriminating tumor regions in cancerous tissues [7,8,9]. Matrix-assisted laser desorption ionization (MALDI) is a widely used ionization method of imaging metabolites and lipids in a variety of biological samples. The progress and applications of MALDI MSI have been summarized in several reviews [10,11,12]. However, MALDI conventionally requires a high vacuum environment, making it impractical to use when analyzing volatile molecules. Moreover, peaks produced by the organic matrices often interfere with peaks of interest in the low *m*/*z* spectra, increasing the difficulty in accurately deciphering the mass spectra [13,14]. These challenges demand the development of advanced MSI instrumentation and methodology operated under ambient conditions, such as atmospheric pressure MALDI (AP-MALDI) [15,16], probe electrospray ionization (PESI) [17,18], desorption electrospray ionization (DESI) [19,20], and infrared matrix-assisted laser desorption ionization (IR-MALDESI) [21,22,23].

IR-MALDESI is a rapid and organic matrix-free MS ionization source combining the benefits of MALDI with post-electrospray ionization (ESI) [24]. In IR-MALDESI experiments, a pulsed 2.97 µm mid-infrared laser resonantly excites the O-H stretching bond of endogenous water and/or an exogenously deposited ice matrix, which desorbs neutral species within the laser spot [25]. The desorbed neutral molecules further partition into small droplets in the orthogonal electrospray and are ionized in an ESI-like mechanism before being introduced into a high resolving-power mass spectrometer [26,27,28]. The current IR-MALDESI source is interfaced with an Orbitrap Exploris 240 mass spectrometer. Over the past decade, IR-MALDESI has advanced significantly in the direct analysis as well as spatially resolved mass spectrometry imaging of biomolecules, including metabolites and lipids [9,29].

One of the challenges facing the MSI field is the variations of ionization efficiency among metabolites due to their structural diversity. While some metabolites are readily detected in positive ionization mode, others like fatty acids (FAs) and glycerophosphates (PAs) are more conducive to analysis in negative ionization mode. Herein, researchers usually conduct two separate MSI experiments in both polarities to improve molecular coverage. However, running two experiments in both ionization polarities is time-intensive and challenging especially when the sample is valuable and limited. Furthermore, MSI analysis in negative ionization mode does not perform as robustly compared to positive ionization mode due to increased tendency for corona discharging [30,31]. To solve these problems and maintain the same metabolite profile, a polarity-switching method is developed such that the electrospray polarity was rapidly alternated in adjacent voxels, which allows for detection of positively and negatively charged species from the same sample in a single experiment; however, the spatial resolution of X dimension is compromised (two times lower in X dimension than Y dimension) [7]. Furthermore, the polarity-switching method is not universally applicable to all mass spectrometers [32,33]. An alternative method named paired-ion electrospray ionization (PIESI) is to add doubly- or multiply-charged reagents into electrospray solvent to form positively charged adducts with deprotonated biomolecules, which allow them to be detected in positive ionization mode. First introduced in 2005 [34], PIESI has been used in liquid chromatography (LC) ESI MS [35,36,37], single probe ESI MS [38,39], and DESI MS [40,41,42] to help detect inorganic anions, deprotonated glycerophospholipids, and other anionic ions. Although the mechanism is still under exploration, previous evidence suggested that binding equilibrium constants, surface activity, and structural flexibility could partly explain the ionization efficiency of ion-pairing reagents [34,43,44].

In this study, we demonstrated the feasibility of a dicationic reagent, 1,5-pentanediyl-bis(1-butylpyrrolidinium) difluoride (abbreviated to [C_5_(bpyr)_2_]F_2_), to detect deprotonated metabolites in positive ionization mode via IR-MALDESI MS. The dicationic reagent [C_5_(bpyr)_2_]^2+^ contains two cationic moieties connected by a hydrocarbon chain. Once [C_5_(bpyr)_2_]^2+^ binds to an anionic ion (−1 charged), the resulting positively charged adducts can be imaged in the positive ionization mode. The use of dicationic reagent [C_5_(bpyr)_2_]^2+^ was reported in many studies for MS analysis [35,36,37,38,39,45] and showed superior performance for analyzing anions in ESI MS [37]. Since the ionization mechanism of IR-MALDESI is similar to ESI [26,27,28], we can ideally expect a similarly superior performance for detecting negatively charged ions in positive ionization mode with IR-MALDESI. Additionally, in a single-probe MSI experiment, [C_5_(bpyr)_2_]^2+^ was added to the solvent and used to extract the chemical contents, which led to the detection of a significant number of deprotonated metabolites in positive ionization polarity [38]. Hence, we chose this representative dicationic reagent [C_5_(bpyr)_2_]^2+^ to enrich the metabolomic coverage for IR-MALDESI MSI. The method development was carried out with quasi-homogeneous rat liver sections. Following that, hen ovary sections were tested as a model system for mass spectrometry imaging. Tandem mass spectrometry (MS/MS) substantiated that adducts were likely formed between deprotonated ions and [C_5_(bpyr)_2_]^2+^. 

## 2. Results and Discussions

### 2.1. Detection of Negatively Charged Metabolites in Adducted Positive Ionization Polarity

**Figure 1:**
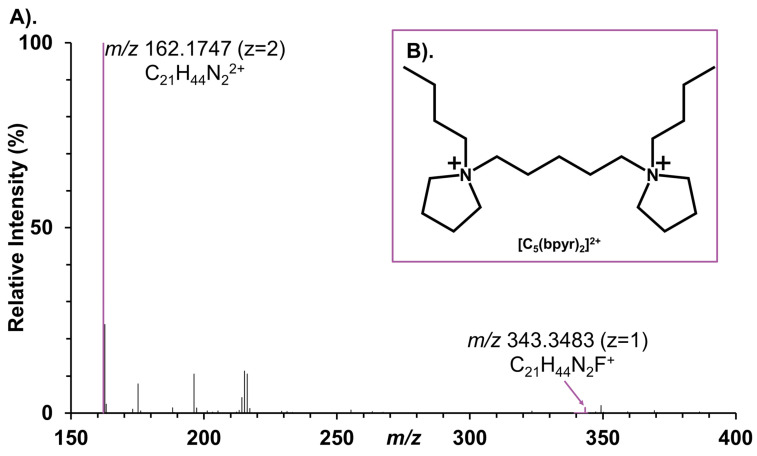
(**A**). Mass spectrum of [C_5_(bpyr)_2_]^2+^ at 10 µM doped in the 50% MeOH/H_2_O (*v*/*v*) electrospray solvent. Peaks related to [C_5_(bpyr)_2_]^2+^ are labeled in purple. (**B**). Dicationic chemical structure of [C_5_(bpyr)_2_]^2+^.

The addition of 10 µM [C_5_(bpyr)_2_]^2+^ into the ESI solvent resulted in the peak at 162.1747 *m*/*z* (z = 2) with an average ion flux of >10^7^ ions/sec in the ESI mass spectrum (Figure 1A). This indicated the dicationic ion was readily detected and can be used for tissue analyses. Rat liver was chosen to study the efficacy of the [C_5_(bpyr)_2_]^2+^ due to its quasi-homogeneous nature and well-studied metabolite profile. We conducted IR-MALDESI MSI analysis of rat liver sections with a low *m*/*z* window (150–600) and a high *m*/*z* window (350–1400) separately in negative ionization, positive ionization, and [C_5_(bpyr)_2_]^2+^ adducted positive ionization mode.

**Figure 2:**
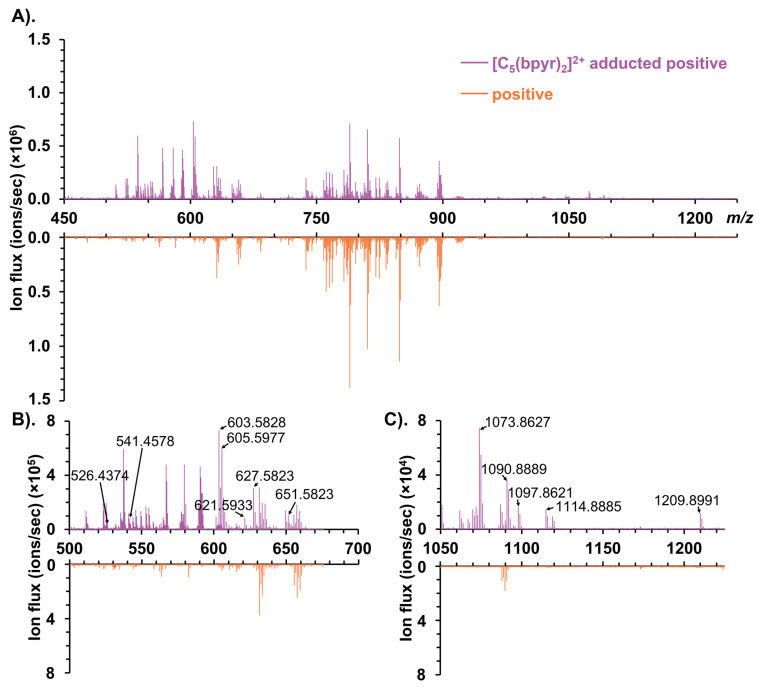
Comparison of mass spectra from rat liver sections between [C_5_(bpyr)_2_]^2+^ adducted positive ionization mode (purple, top) and positive ionization mode (orange, bottom); (**A**). Full mass spectrum at 450–1250 *m*/*z*; (**B**,**C**). Zoomed-in mass spectra with labeled *m*/*z* related to adducts with [C_5_(bpyr)_2_]^2+^.

**Figure 3:**
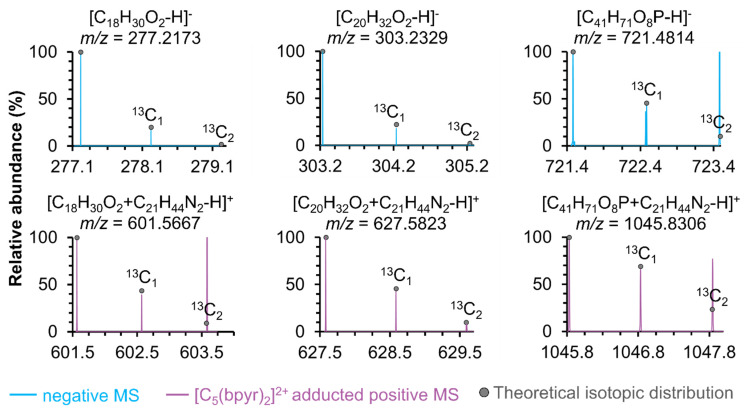
Spectral accuracy determination of three representative deprotonated metabolite ions in negative ionization mode (top, blue) and [C_5_(bpyr)_2_]^2+^ adducted positive ionization mode (bottom, purple), respectively. All background peaks were removed to clearly show the good alignment between the experimental MS spectra (shown in lines) and theoretical ^13^C isotopic distribution (marked in grey dots).

In negative ionization mode, most negatively charged metabolites were detected in the low *m*/*z* window, while a distinct cluster containing deprotonated glycerophospholipids, such as PAs, was observed between 700 and 950 *m*/*z* (Figure S1). Furthermore, we compared the mass spectrum in positive ionization mode to that in [C_5_(bpyr)_2_]^2+^ adducted positive ionization mode (Figure 2). We found three noticeable mass spectral features stood out in the *m*/*z* range of 500–675, 750–900, and 1000–1250, respectively. Figure 2A displays that the abundant species normally observed in positive ionization mode, such as Glycerophosphocholines (PCs) and Glycerophosphoethanolamines (PEs), still dominated at 750–900 *m*/*z* while using [C_5_(bpyr)_2_]^2+^. Detailed spectra at 500–675 *m*/*z* (Figure 2B) and 1000–1250 *m*/*z* (Figure 2C) exhibited that a few peaks were only observed in [C_5_(bpyr)_2_]^2+^ adducted positive ionization mode, therefore, they were putatively assigned as the potential adduct ions with [C_5_(bpyr)_2_]^2+^. Each *m*/*z* value of those peaks was subtracted by the mass shift of 324.3494 (dicationic mass of [C_5_(bpyr)_2_]^2+^). The resulting *m*/*z* values were searched against the METLIN database with the parameter settings of the negative charge, [M-H]^-^ and 2.5 ppm mass measurement accuracy (MMA). In addition to high mass measurement accuracy, spectral accuracy is the instrument’s ability to accurately measure the isotopic distribution. Combing high spectral accuracy with high MMA, we can determine the elemental composition of one compound based on its isotopic distribution [46]. In this work, the number of carbons in each molecule was estimated by dividing the relative abundance of the M + 1 peak containing one ^13^C by the natural isotopic abundance of ^13^C (~1.11%). As Figure 3 was shown, there was good agreement between experimental and theoretical isotopic abundance, increasing our confidence in the putative identifications.

**Figure 4:**
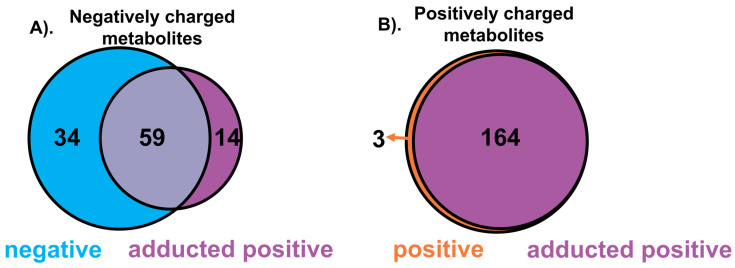
Venn diagram summarizing the number of metabolites from rat liver sections tentatively identified in negative, positive, and [C_5_(bpyr)_2_]^2+^ adducted positive ionization modes, respectively. (**A**). Negatively charged metabolites detected in negative ionization and [C_5_(bpyr)_2_]^2+^ adducted positive ionization modes. (**B**). Positively charged metabolites detected in positive ionization and [C_5_(bpyr)_2_]^2+^ adducted positive ionization modes. All putative identifications were made via accurate *m*/*z* matching in the METLIN database within 2.5 ppm MMA. Detailed information regarding putative identifications, molecular formula, and categories are listed in Tables S1–S3.

Overall, we found 73 deprotonated metabolites formed adduct ions with [C_5_(bpyr)_2_]^2+^, among which 59 were also detected in negative ionization mode (Figure 4A, refer to Tables S1 and S2 for the detailed list of metabolites). Most of them were manually classified into FAs with a few PAs and PEs species according to LIPID MAPS. The number of positively charged metabolites observed in positive ionization mode from the rat liver section was 167; 164 were still observed at their usual *m*/*z* when [C_5_(bpyr)_2_]^2+^ was added to ESI solvent (Figure 4B, refer to Table S3 for the detailed list of metabolites). The main classes for most positively charged metabolites were PCs, PEs, Diradylglycerols (DGs), and Triradylglycerols (TGs).

### 2.2. Mass Spectrometry Imaging of a Hen Ovary Section with the Dicationic Reagent

**Figure 5:**
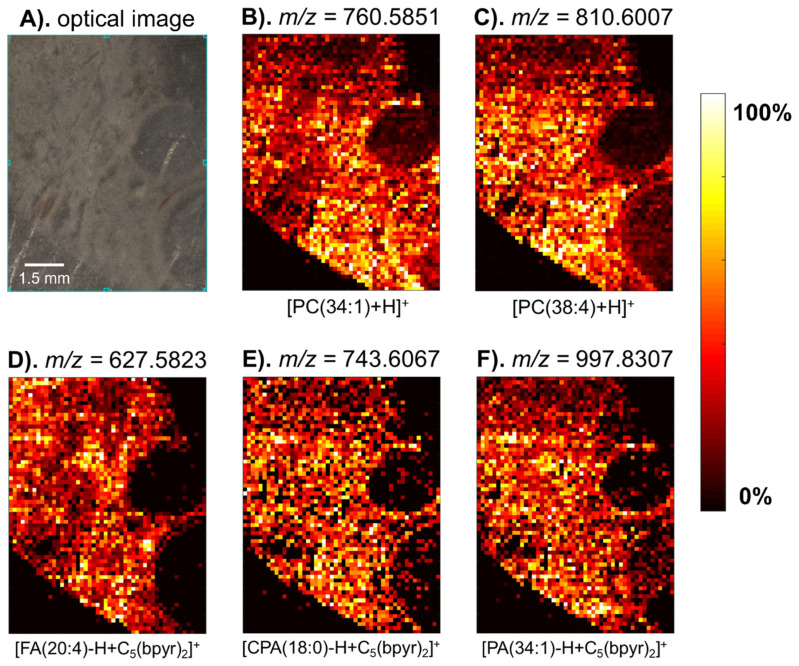
Heatmap images of metabolites detected from the hen ovary section in [C_5_(bpyr)_2_]^2+^ adducted positive ionization polarity with their putative identifications labeled at the bottom. (**A**). Optical image of the hen ovary section before MS analysis. (**B**,**C**). Heatmaps of representative protonated metabolites. (**D**–**F**). Heatmaps of representative deprotonated metabolites adducted with [C_5_(bpyr)_2_]^2+^.

Following the method development, the hen ovary tissue sections were chosen for mass spectrometry imaging as the sample contained more morphological features. The desorbed neutrals from the hen ovary tissue sections were post-ionized by ESI solvent containing [C_5_(bpyr)_2_]^2+^. The *m*/*z* range was adjusted to 200–1000 for negative ionization polarity to encapsulate the most negatively charged metabolites we observed in the rat liver results. In positive and [C_5_(bpyr)_2_]^2+^ adducted positive ionization modes, the mass range was altered to 350–1400 *m*/*z*. Two main reasons for this change were (1) to include a majority of [C_5_(bpyr)_2_]^2+^ adduct ions between 525 and 1325 *m*/*z*; (2) to retain a few positively charged metabolites that were normally seen between 350 and 500 *m*/*z*. Since the goal of this part of the study is to assess the capability of the [C_5_(bpyr)_2_]^2+^ to enrich the molecular profile of metabolomic mass spectrometry imaging instead of imaging the whole hen ovary section, we chose to only sample a small ROI that contained two follicles (Figure 5A). In total, 18 deprotonated metabolites were able to form adducts with [C_5_(bpyr)_2_]^2+^ in the adducted positive ionization mode (refer to Table S4 for the detailed list of metabolites). Two heatmaps of representative protonated lipid ions were shown along with three adducted ions in Figure 5B–F. The heatmaps exhibited the same spatial distribution and were highly correlated with the morphological features displayed in the optical image.

### 2.3. MS/MS Analysis of Selected Lipid Adducts with the Dicationic Reagent

**Figure 6:**
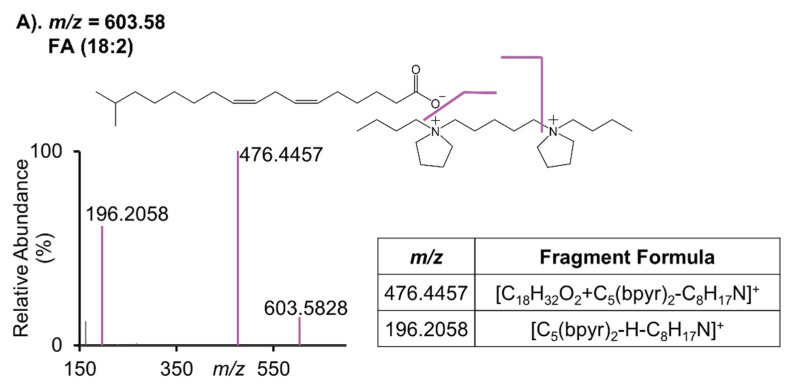

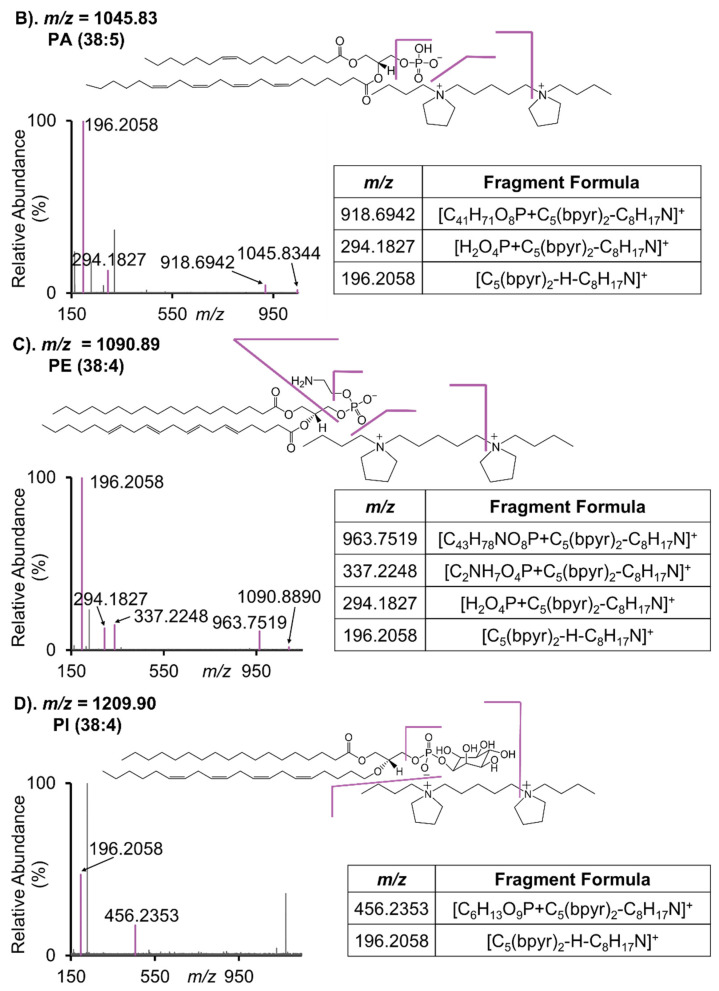
MS/MS mass spectra of selected adducted ions with their putative identifications and structures. (**A**). [FA(18:2)-H+C_5_(bpyr)_2_]^+^; (**B**). [PA(38:5)-H+C_5_(bpyr)_2_]^+^; (**C**). [PE(38:4)-H+C_5_(bpyr)_2_]^+^; (**D**). [PI(38:4)-H+C_5_(bpyr)_2_]^+^, noting that the fragment peak at 1082.7628 *m*/*z* was not seen in the spectra possibly due to the low abundance of its precursor ion at 1209.8991 *m*/*z*.

To acquire more information about the adduct formation and to validate the identifications of adducted ions, we conducted HCD MS/MS experiments on selected adducted ions from the rat liver tissue section. A common fragment ion at *m*/*z* 196.2058 was detected in all MS/MS mass spectra (Figure 6A–D), which is derived from [C_5_(bpyr)_2_]^2+^ itself losing C_8_H_17_N (127.1362 Da). The same neutral loss of 127.1362 Da was also observed in the fragment ions detected at 476.4457 *m*/*z* in Figure 6A, at 918.6942 *m*/*z* in Figure 6B, and at 963.7519 *m*/*z* in Figure 6C, respectively. However, in Figure 6D, we did not observe either the precursor ion at 1209.8991 *m*/*z* or the expected fragment ion at 1082.7628 *m*/*z* (i.e., the fragment ion caused by the neutral loss of 127.1362 Da), possibly due to the low abundance of the precursor ion at 1209.8991 *m*/*z* (with an averaged ion flux ~10^4^ ions/sec). Importantly, we found a fragmentation scheme [headgroup of glycerophospholipid+ C_5_(bpyr)_2_-C_8_H_17_N]^+^, i.e., for PA, [H_2_O_4_P+C_5_(bpyr)_2_-C_8_H_17_N]^+^ at 294.1823 *m*/*z* in Figure 6B; for PE, [C_2_NH_7_O_4_P+C_5_(bpyr)_2_-C_8_H_17_N]^+^ at 337.2248 *m*/*z* in Figure 6C; for PI, [C_6_H_10_O_8_P+C_5_(bpyr)_2_-C_8_H_17_N]+ at 456.2353 *m*/*z* in Figure 6D. As Figure 6A–D shown, the peaks related to [headgroup of glycerophospholipid+C_5_(bpyr)_2_-C_8_H_17_N]^+^ and [lipid+C_5_(bpyr)_2_-C_8_H_17_N]^+^ were retained, whereas the commonly occurring product peaks for lipid identifications were not detected. One possible explanation for this phenomenon is that the anionic lipids formed ionic bonds with [C_5_(bpyr)_2_]^2+^. Specifically, when the lipids were glycerophospholipids such as PAs, PEs, and PIs, the ionic bonds were built between their headgroup and one of the cationic moieties of the dicationic reagent. Given that the electrostatic force between ionic bonds is generally stronger than that between covalent bonds, the covalent bonds in the adducted ions were cleaved first. The fragmentation pattern is very interesting and warrants further investigation. This could lead us to explore specific types of dicationic reagents regarding their cationic moieties to enhance the detection of glycerophospholipids as well as other categories of lipids and metabolites. It is also important to point out that the fragment peak at 294.1823 *m*/*z* that was observed in Figure 6B was also found in Figure 6C, which could be due to a neutral loss of C_2_NH_5_ (43.0422 Da) from the headgroup of PEs.

One possible hypothesis that could explain the adduct formation with dicationic reagent is that this chemistry is happening on a millisecond scale at the surface of the droplet. After the mid-infrared laser irradiates the tissue sample, neutral species are ejected and encounter the orthogonally oriented electrospray plume where they are ionized in an ESI-like process [26,27,28]. Since all charges are located on the droplet surface, [C_5_(bpyr)_2_]^2+^ and other cationic ions (e.g., H^+^) are competing against each other for the adduct formation simultaneously. Therefore, we hypothesize that [C_5_(bpyr)_2_]^2+^ outcompetes others due to two possible reasons: (1) the binding equilibrium constant for [C_5_(bpyr)_2_]^2+^ adducts is greater than the binding equilibrium constants for other possible adducts. As a result, neutral species that are liable to get deprotonated could be more easily adducted with [C_5_(bpyr)_2_]^2+^; (2) it is more likely that metabolites form ionic bonds with [C_5_(bpyr)_2_]^2+^. Since the electrostatic force within an ionic bond is generally stronger than that of a covalent bond, it is easier to form an ionic bond between oppositely charged ions (i.e., neutral species that can be negatively ionized and [C_5_(bpyr)_2_]^2+^ that is +2 positively charged, respectively).

## 3. Materials and Methods

### 3.1. Materials

The dicationic reagent 1,5-pentanediyl-bis(1-butylpyrrolidinium) difluoride ([C_5_(bpyr)_2_]F_2_) at the concentration of 2.5 mM in 50% methanol/H_2_O (*v*/*v*) was purchased from AZYP, LLC (Arlington, TX, USA). The dicationic structure was observed at 162.1747 *m*/*z* (z = 2) and 343.3483 *m*/*z* (z = 1) with high ion abundance (Figure 1A). The dicationic chemical structure was shown in Figure 1B. [C_5_(bpyr)_2_]F_2_ was diluted to 10 µM with 50% methanol/H_2_O in this experiment. LC/MS grade methanol, formic acid, and water were purchased from Fisher Chemical (Fair Lawn, NJ, USA). Nitrogen gas for purging the enclosure was purchased from Arc3 Gases (Raleigh, NC, USA).

### 3.2. Preparation of Rat Liver and Hen Ovary Sections

Rat liver and domestic hen ovary tissues were provided from the NCSU Department of Biological Sciences and the Prestage Department of Poultry Science, respectively, and stored at −80 °C before the IR-MALDESI analysis. The utilized animal tissues were managed in accordance with the Institute for Laboratory Animal Research Guide. All husbandry practices were approved by the North Carolina State University Institutional Animal Care and Use Committee (IACUC, Raleigh, NC, USA). The tissues were sectioned to 15 µm using a Leica CM1950 cryostat (Buffalo Grove, IL, USA) at −15 °C and then thaw-mounted onto microscope glass slides.

### 3.3. IR-MALDESI Experimental Parameters

The home-built IR-MALDESI source was elaborately described in previous publications [47,48]. The “burst-mode” 2.97 µm infrared laser was developed by JGM Associates, Inc. (Burlington, MA, USA) and set to 10 pulses/burst to generate ~1.0 mJ/burst laser energy for this experiment [49]. An ice layer was exogenously deposited on top of the sample by purging the enclosure with dry nitrogen to achieve a relative humidity of <12% and cooling down the stage to −8 °C before exposing the sample to the ambient environment for several minutes to promote ice growth. Then, the enclosure was closed and purged again to maintain a relative humidity of <12% throughout each experiment. The electrospray solvent was 50% MeOH/H_2_O (*v*/*v*) modified with 0.2% formic acid for both positive and negative ionization modes. For [C_5_(bpyr)_2_]^2+^ adducted positive ionization mode, the dicationic reagent was added into the electrospray solution at 10 µM. The ESI solution was delivered by a syringe pump (Fusion 101, Thermo Fisher Scientific, Bremen, Germany) at a flow rate of 1.5 µL/min. The IR-MALDESI source is currently coupled to a high resolving power Orbitrap Exploris 240 mass spectrometer (Thermo Fisher Scientific, Bremen, Germany). The instrument was set to “Small Molecules” mode with a resolving power of 240,000_FWHM_ at 200 *m*/*z*. To coordinate the laser desorption and ion acquisition events, the automatic gain control function (AGC) was disabled, and the optimal injection time was set to 15 ms for both MS and MS/MS analyses. The raster step size was set to 150 µm. The pixel size was 150 × 150 µm.

For the method development on rat liver sections, MS data were collected across two separate *m*/*z* ranges to allow for broader coverage and to ensure inclusion of the adducted species given the increase in mass shift: 150–600 *m*/*z* and 450–1800 *m*/*z* for all conditions (i.e., negative ionization, positive ionization, and [C_5_(bpyr)_2_]^2+^ adducted positive ionization). The region-of-interest (ROI) was 20 × 20 scans on tissue and 10 × 10 scans off tissue. Each condition was repeated three times. For imaging hen ovary sections, the *m*/*z* ranges were adjusted to 200–1000 for negative ionization mode, and 350–1400 for both positive and [C_5_(bpyr)_2_]^2+^ adducted ionization mode to include most cationic metabolites, as well as adducted ions. MS/MS analyses of selected adducted ions from rat liver tissue sections were conducted using higher-energy collision-activated dissociation (HCD) with normalized collision energy (NCE) of 50%. The isolation window was set to 1.0 or 1.5 Da based on the *m*/*z* of the precursor ions.

### 3.4. Data Analysis

All the Xcalibur raw files were first converted into mzML format using the Mscovert from the ProteoWizard software package [50] and further converted to imzML [51] format by an imzML converter [52]. The imzML files were loaded into MSiReader version 1.02 [53,54] (available at https://msireader.ncsu.edu/ (accessed on 2 September 2021)) operated in the MATLAB environment (R2019b; MathWorks, Natick, MA, USA). The MSiPeakfinder tool was used to generate potential tissue-specific ions with mass measurement accuracy (MMA) of ±2.5 ppm, which showed 1.5× or higher abundance ratios than the off-tissue area. The putative metabolite identification of potential tissue-specific ions was carried out by searching *m*/*z* in METLIN [55] (https://metlin.scripps.edu/index.php (accessed on 10 September, 2021)). Imaging data obtained from hen ovary sections were also subject to METASPACE [56] (https://metaspace2020.eu/ (accessed on 25 August 2021)) and metabolites were putatively annotated with a 10% false discovery rate using HMDB-v4. Most putatively identified metabolites were lipids, which were manually categorized based on LIPID MAPS (https://www.lipidmaps.org/data/classification/LM_classification_exp.php (accessed on 16 November 2021)).

## 4. Conclusions

In this work, the deprotonated metabolites commonly seen in negative ionization mode were observed in positive ionization mode through adduct formation when doping [C_5_(bypr)_2_]^2+^ in the electrospray solvent. By utilizing this method, we enhanced the metabolite coverage obtained in the positive ionization mode within the same *m*/*z* range without the need to run two experiments in both ionization polarities, which saved data acquisition time and reduced sample consumption. This method was tested on rat liver sections and hen ovary sections, revealing that it has the potential to be used with different biological samples. Tandem mass spectrometry presented evidence that the dicationic reagent [C_5_(bypr)_2_]^2+^ was likely attracted to the headgroup of glycerophospholipid ions. In future work, we will optimize the dicationic reagent concentration to maximize the number and ion abundances of specific kinds of negatively charged metabolites, which could provide more biological depth in a single MSI analysis.

## Data Availability

Data are available within the article and Supplementary Material online.

## Supplementary Materials

The following supplemental materials are available online at https://www.mdpi.com/article/10.3390/metabo11120810/s1. Figure S1: The representative mass spectrum was obtained in negative ionization mode from the rat liver section, Table S1: 93 negatively charged metabolites from the rat liver section detected in negative ionization mode, Table S2: 73 negatively charged metabolites from the rat liver section forming adducted ions with the dicationic reagent detected in adducted positive ionization mode, Table S3: 167 positively charged metabolites from the rat liver section detected in positive ionization mode, Table S4: 18 negatively charged metabolites from the hen ovary section forming adduct ions with the dicationic reagent detected in adducted positive ionization mode.
