# Supplementary material for: Enhancing Metabolomic Coverage in Positive Ionization Mode Using Dicationic Reagents by Infrared Matrix-Assisted Laser Desorption Electrospray Ionization
Source: Metabolites. 2021 Nov 29;11(12):810. doi: 10.3390/metabo11120810 (PMC8708802; doi:10.3390/metabo11120810)
Supplement: Supplementary file 1 [file metabolites-11-00810-s001.zip › Supplementary File_1_FigureS1.pdf]

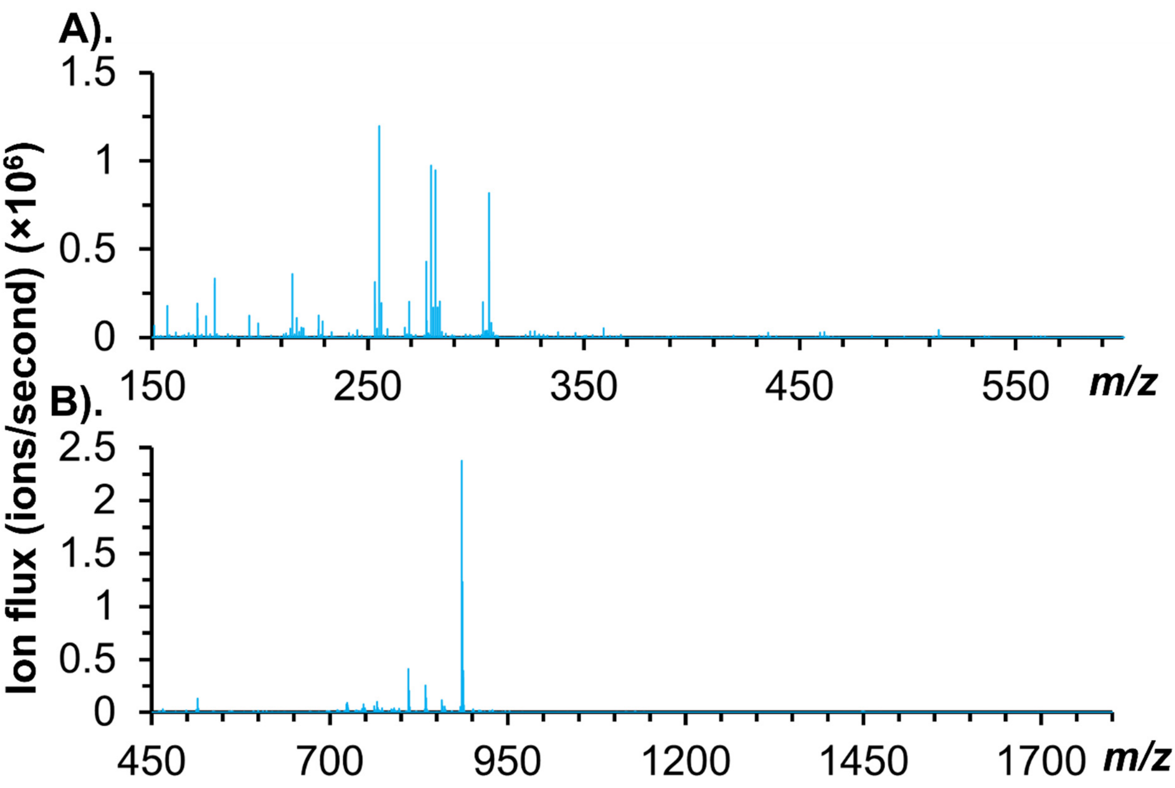

Figure S1. The representative mass spectrum was obtained in negative ionization mode from the rat liver section. **A).** at 150-600 *m/z*; **B).** at 450-1800 *m/z*.
